# A School Meals Program Implemented at Scale in Ghana Increases Height-for-Age during Midchildhood in Girls and in Children from Poor Households: A Cluster Randomized Trial

**DOI:** 10.1093/jn/nxz079

**Published:** 2019-05-17

**Authors:** Aulo Gelli, Elisabetta Aurino, Gloria Folson, Daniel Arhinful, Clement Adamba, Isaac Osei-Akoto, Edoardo Masset, Kristie Watkins, Meena Fernandes, Lesley Drake, Harold Alderman

**Affiliations:** 1 International Food Policy Research Institute, Washington, DC; 2 Partnership for Child Development, Department of Infectious Disease Epidemiology, Imperial College, London, London, United Kingdom; 3 Noguchi Memorial Institute for Medical Research, College of Health Sciences; 4 Institute of Statistical, Social, and Economic Research, University of Ghana, Legon, Ghana; 5 Institute of Development Studies, University of Sussex, Brighton, United Kingdom

**Keywords:** school age, adolescence, nutrition, school meals, impact evaluation

## Abstract

**Background:**

Attention to nutrition during all phases of child and adolescent development is necessary to ensure healthy physical growth and to protect investments made earlier in life. Leveraging school meals programs as platforms to scale-up nutrition interventions is relevant as programs function in nearly every country in the world.

**Objective:**

The aim of this study was to evaluate the impact of a large-scale school meals program in Ghana on school-age children's anthropometry indicators.

**Methods:**

A longitudinal cluster randomized control trial was implemented across the 10 regions of Ghana, covering 2869 school-age children (aged 5–15 y). Communities were randomly assigned to *1*) control group without intervention or *2*) treatment group providing the reformed national school feeding program, providing 1 hot meal/d in public primary schools. Primary outcomes included height-for-age (HAZ) and BMI-for-age (BAZ) *z* scores. The analysis followed an intention-to-treat approach as per the published protocol for the study population and subgroup analysis by age (i.e., midchildhood for children 5–8 y and early adolescence for children 9–15 y), gender, poverty, and region of residence. We used single-difference ANCOVA with mixed-effect regression models to assess program impacts.

**Results:**

School meals had no effect on HAZ and BAZ in children aged 5–15 y. However, in per-protocol subgroup analysis, the school feeding intervention improved HAZ in 5- to 8-y-old children (effect size: 0.12 SDs), in girls (effect size: 0.12 SDs)—particularly girls aged 5–8 y living in the northern regions, and in children aged 5–8 y in households living below the poverty line (effect size: 0.22 SDs). There was also evidence that the intervention influenced food allocation and sharing at the household level.

**Conclusion:**

School meals can provide a platform to scale-up nutrition interventions in the early primary school years, with important benefits accruing for more disadvantaged children. This trial was registered at www.isrctn.com as ISRCTN66918874.

## Introduction

Attention to nutrition during all phases of child and adolescent development is necessary to ensure healthy development over the 8000 d spanning infancy to adulthood, and to protect investments made earlier in the life course (1). Although there are relatively few investments proven to be cost-effective at scale after the first 1000 d (2), preschool and school-based programs may be a practical platform to reach children and adolescents at scale. Although less cost-effective for addressing undernutrition than early interventions (3), school feeding—or school meals—is a multisectoral intervention with impacts across education, health and nutrition, and food security that is widely implemented; globally, programs reach ∼368 million children for a total investment of ∼$70 billion a year (4). Rigorous studies have shown that school feeding can improve school attendance and learning, as well as a child's physical and psychosocial health [see Kristjansson et al. (5) for a systematic review]. These effects are heterogeneous and context-specific, depending on the economic environment as well as on the quality of implementation. There is a paucity of evidence, however, on government-led programs at scale, where implementation constraints may be critical.

Furthermore, most of the studies on school feeding predate the substantial progress in school enrollment in recent years; net primary enrollment increased globally from 83% in 1999 to 90% in 2016 (6). Low-income countries are approaching universal primary enrollment, which improves the potential of school-based health and nutrition programs, such as school feeding, to reach large proportions of children and adolescents. Concurrent with changes in enrollment goals, the objective of improving nutrition has shifted in recent years as many countries see school meals as a means to address the challenge of obesity, rather than primarily to offset undernutrition. There is a need then to understand school meal programs’ distribution of benefits across populations—particularly the most vulnerable groups—and, where apparent, of nutritional risk.

This study is aimed at addressing these evidence gaps by evaluating the impact of the national school feeding program in Ghana, focusing on primary outcomes relevant to nutrition, namely height-for-age *z* scores (HAZs) and BMI-for-age *z* scores (BAZs); the results for the education and agriculture analysis will be published separately.

### Nutrition and growth in school-age children

**FIGURE 1:**
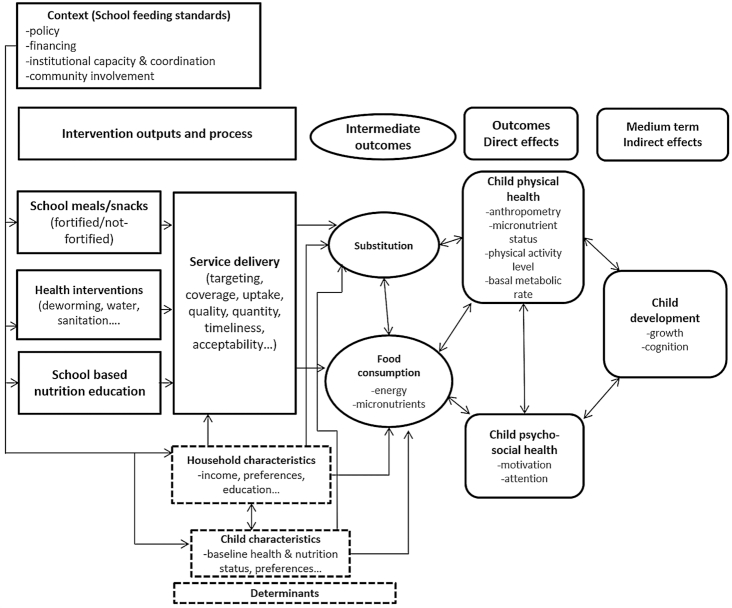
Program impact pathways for the school feeding intervention, including anthropometry as an indicator of child physical health.

Although not the sole determinant of nutritional status, food consumption, in terms of quantity, quality, and diversity, plays a major role in determining nutritional status and provides a pathway linking school feeding to nutrition outcomes (**Figure 1**). School feeding is generally designed to supplement food provided at home and improve schoolchildren's food intake. However, school food could be shared by schoolchildren with other household members or substitute for food normally consumed at home. This is in most cases planned for in take-home-ration interventions, in which children take home a quantity of food on a regular basis, with some being consumed by other family members or sold (7). This also applies to any school feeding program because households may in principle use the school meal as a substitute for food normally consumed at home and spend the monetary equivalent otherwise. If children benefitting from school feeding are malnourished, substitution within households is ambiguous; it could reduce potential nutritional benefits to the school-going child, but it could also benefit her siblings. The evidence on reallocation in households receiving school meals indicates that most of the energy provided by the program “sticks” with the beneficiaries (8, 9). However, there is also evidence that school meals programs can enhance the nutrition status of younger siblings of students (10). There also could be a trade-off where providing energy and micronutrients to stunted children through school meals could result in adding weight rather than height, thus contributing to increasing overweight and obesity. More broadly, beyond the role of a food transfer, the school food environment may provide an entry point to support nutrition and health in schoolchildren (11). Research in high-income countries highlights the role of school feeding, food advertising, nutrition education, sales of snacks and beverages, and peer influences in shaping behaviors (12, 13). Less is known about these issues in low- and middle-income countries (11).

## Methods

### Country context

Ghana is a lower-middle-income country of 25 million people situated in West Africa, with >40% of its population aged <15 y (14). Ghana is ranked 140th in the 2018 Human Development Index table, with life expectancy at birth of 63 y, expected schooling of 11.6 y, and gross national income per capita of $4096 (6). Approximately 25% of the population is estimated to live in poverty based on the national-level poverty line. The prevalence of malnutrition in young children in Ghana has been assessed through the Ghana Demographic and Health Surveys conducted every 5 y since 1988. From 2003 to 2014, stunting in children <5 y of age decreased from 35% to 19% (15). Evidence on school-age children in Ghana is scarce and limited to small sample studies. A cross-sectional study of 100 randomly selected upper-primary-school children from 5 schools in Tamale, a major urban center in Northern Ghana, found the prevalence of underweight was 10%, whereas 7% were at risk of becoming overweight and 4% were overweight (16). Another cross-sectional study investigated dietary intakes and nutritional status of 182 school-age children participating in 2 semirural communities and found that 48% were stunted, 35% had low BAZ, and 1% was overweight (17). Another study exploring malnutrition among school-age children in the Volta Region found that, among 650 randomly selected children between 10 and 19 y, the prevalence of overweight was 7%, stunting 50%, and thinness 19% (18).

### The intervention

In 2015, the Ghana School Feeding Program (GSFP) reached >1.6 million primary-school children across the 170 districts of the country (19). Funded entirely by the Government of Ghana, the program has a 4-y budget of >$200 million. The GSFP is designed as a multisectoral strategy to increase food production, household income, and food security in deprived communities (20), integrating child-level education and nutrition with household agriculture and social-protection objectives. The implementation of the GSFP is managed through a National Secretariat, with oversight provided by the Ministry of Gender, Children and Social Protection. The school meals service is provided through caterers contracted directly by the GSFP based on a 40 Ghana pesewas ($0.33) allocation per child per day. Each caterer is responsible for buying food from markets and preparing and distributing the meals in schools. Cash is transferred to caterers retrospectively covering a 2-wk period. Caterers are not allowed to serve >3 schools and their profits are made on margins after food procurement, preparation, and distribution. The school meal menus are designed at the district level to meet ∼30% of the recommended daily intake for children aged 6–12 y (21), and include foods grown by farmers in the community and the broader agroecological zone (22). School-level supervision on the quality of the service provision is provided by the School Implementing Committee. A supply chain study of the GSFP reported that the main challenges faced by caterers included changes in food prices, and the inability to mitigate price fluctuations because of payment delays (23). Price variations between harvest and lean seasons as reported by caterers involved increases of ≤400%. Because payments from the GSFP are retrospective, caterers were often found to not have the resources to buy in bulk at lower prices. Caterers also reported buying on credit from market traders, thus weakening their negotiation position. Caterers also highlighted that payments and budgets did not reflect the actual numbers of children served, as enrollment tended to increase during the school year, resulting in higher costs for caterers. Caterers responded to these challenges by adapting the menus, reducing portion sizes, or adjusting the quality of the food (23).

### Study design and participants

A cluster randomized control trial (CRCT) (ISRCTN66918874) was designed around the scale-up of the Ghana School Feeding Program (GSFP) across the 10 regions of Ghana. For the study protocol details, see Gelli et al. (20). Briefly, the GSFP set clear criteria for the selection of the intervention areas as captured in the retargeting exercise conducted in 2012. Poverty rankings were developed using the Ghana Living Standards Survey and the Core Welfare Indicators Questionnaire carried out in 2005/2006 and 2003, respectively. Food consumption scores were calculated using the Comprehensive Food Security and Vulnerability Assessment 2008/2009 and spatial data variables computed by the World Food Programme (24). The data were used to generate district-level composites for share of national poverty and food insecurity that were used to allocate program resources. As a result, >70% of the government investment in the national program reaches the poorest areas of the country (25).

### Randomization

**FIGURE 2:**
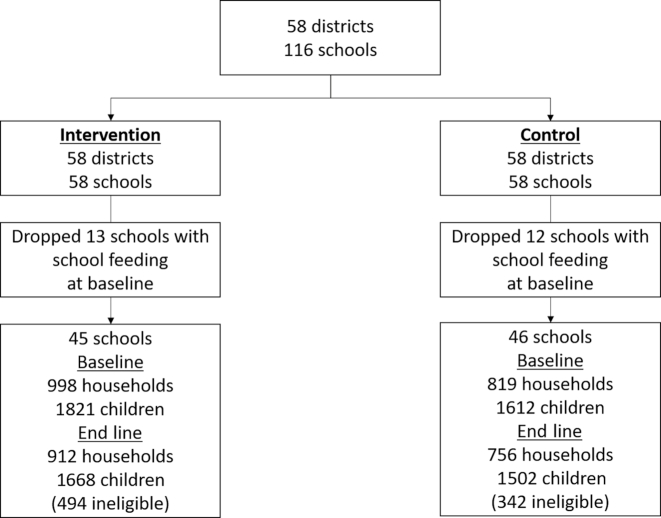
Schematic view of the randomization process and trial profile.

Households and schools were randomly assigned to 2 treatment arms (**Figure 2**):

1. Control group: schools and households from communities in which the intervention was not implemented for the study duration.
2. Intervention (GSFP) group: schools and households from surrounding communities in which the school feeding program is implemented, with caterers responsible for food procurement and preparation.

Selection of the study areas involved 2 key steps. *1*) We selected 58 districts at random within Ghana from a sample frame including all districts in the country. The sample frame was stratified by region and district inclusion was prioritized using data from the GSFP retargeting exercise including data on the prevalence of poverty and food insecurity. *2*) We identified 2 comparable schools within each of the 58 selected districts. A protocol was designed to ensure comparability between schools based on data from the Education Management Information System and minimize the potential for contamination and crossover between the schools and pupils in each district. This step utilized a list from the GSFP secretariat of schools not currently covered by the GSFP in each district. Data from the annual school census from 2011–2012 were then used to match schools not receiving the GSFP and identify the “best matched” pairs, including data on enrollment, gender ratio, classroom numbers and infrastructure conditions, accessibility, and nongovernmental organization support, among other indicators. These characteristics were selected based on the indicators that could affect the functioning of the intervention. The allocation into school feeding and control schools was then randomized within each pair, using a program written by AG using Stata (StataCorp). Survey enumerators were not blinded to the allocation.

Power calculations were undertaken using data from the 2008 Ghana Demographic and Health Survey, where the mean ± SD HAZ of rural children <5 y was −1.03 ± 1.57 and the intracluster correlation coefficient was 0.08. The results of the power calculations and resource availability suggested the adoption of a sample of 25 households from the communities in the catchment areas of the 58 schools receiving the intervention and of 20 households in the communities of the 58 control schools, allowing for the detection of effect sizes of 0.2 SDs at end line. The study targeted all school-age children aged 5–15 y at baseline in the 116 communities. Households were randomly selected for the survey interviews from a household census in the catchment areas of the targeted schools. For details on the sampling procedures, see the study protocol (20). Eligibility was determined based on being of the target age at baseline (5–15 y), or on not being already enrolled in secondary school or in the last grade of primary school (grade 6) at baseline. Ineligible children were dropped from the analysis sample after the end line.

The primary study outcomes per protocol included HAZ and BAZ. Height-for-age is generally used to assess chronic malnutrition in populations of children <5 y of age. BMI has been used to measure nutrition status in adults since the 1960s and more recently throughout childhood, mostly in the context of overweight and obesity. Height-for-age reflects the cumulative effects of insults during a child's life and may thus be less sensitive than BAZ to current circumstance. Although the target age group included all primary school–age children (aged 5–15 y at baseline), the per-protocol subgroup analysis included dividing the school-age population (5–15 y) into midchildhood (5–8 y) and early adolescence (9–15 y) to explore potential heterogeneities of impact by age.

### Data collection

The baseline and end line surveys were undertaken in June, 2013 and March, 2016, respectively. The 2 rounds of surveys included school-, caterer-, household-, and child-level data collection. The household questionnaire included modules on demographic characteristics, farm assets, economic activities, expenditure, farming and other income, anthropometry for all children aged >2 y, and a range of education indicators for all children aged 5–15 y. Anthropometry measurements were undertaken for all children aged 2–15 y during the household interviews at baseline, although at end line measurements were undertaken in primary school–age children only. Height was measured to the nearest 0.1 cm using portable stadiometers (Leicester Height Measures) and weight was measured using electronic scales (Tanita WB-100A/WB-110A Remote Display Version scales). All enumerators collecting anthropometric data were trained using standard WHO guidelines and measurements were practiced before the survey through standardization exercises. From these standardization sessions, inter- and intraobserver variation of measurement error were documented and the necessary corrections to procedures were made. Data on the school feeding program provision (receipt of free school meals, number of days meals were provided over the previous 5 school days) were collected through child-level recall during the household interviews. Structured interviews were also undertaken with the caterer providing the school meal service in each of the targeted schools. The caterer questionnaire included modules on the school meal service provision, including food sourcing, menus and food quantities provided to students, as well as information on the level of education, training, and supervision the caterers received.

The survey enumerators were recruited by the Noguchi Memorial Institute for Medical Research and the Institute of Statistical, Social and Economic Research at baseline and end line, respectively. At baseline, each team led by a supervisor and assisted by community leaders conducted household listings and sampling in each enumeration area (EA). Maps were obtained for most of the EAs from the Ghana Statistical Service. The EA maps made it possible to identify all dwelling structures within a geographical space with a well-defined boundary. All dwelling structures within each EA were serially numbered to facilitate the complete listing of households. The list of households in each EA constituted the sampling frame from which participating households were selected at random for interviews. All questionnaires were checked in the field for consistency and completeness by field supervisors before data entry. Data were entered in Cspro (United States Census Bureau) and later transferred to Stata version 13 for data cleaning and analysis. The HAZ and BAZ of school-age children were calculated using the WHO AnthroPlus software Stata macro based on the 2007 WHO reference for children aged 5–19 y. This is based on the 1977 National Center for Health Statistics/WHO reference, based on a nonobese sample with expected heights from the US population (26). Total household expenditure was estimated as the value (in Ghanaian cedis) of household food and nonfood consumption; poverty status was calculated by comparing per-capita expenditures with the national poverty line [set at 1314 Ghanaian cedis per capita (27)].

### Statistical analysis

The analysis followed an intention-to-treat approach for the study population. Thus, the results reported cover both the students who participated in the program as well as those who were eligible but either went to alternative private schools or else dropped out of schooling. We also include subgroup analysis by age, gender, household poverty, and region of residence as described in the published protocol (20). The subgroup analysis by age involved dividing the school-age population (5–15 y) into midchildhood (5–8 y) and early adolescence (9–15 y). The impact on HAZ and BAZ was estimated using a single-difference ANCOVA model using multilevel regression models accounting for the hierarchical nature of the data (28). The single-difference model specification has the following form:

(1) \begin{equation*}
{Y_{i1}} = {\beta _0}\ + {\beta _1}{T_i} + {\beta _2}{Y_{i0}} + {\varepsilon _i}
\end{equation*}

where ${Y_{i0}}$ is the outcome variable at baseline for the *i*th child, ${Y_{i1}}$is the outcome variable at end line, and ${T_i}$ is a dummy variable for the treatment assignment. The ANCOVA estimator has been shown to provide more efficient estimates of program impact than a difference-in-difference estimator when autocorrelation of outcomes is low (29). The multilevel models included random intercepts at cluster (school) and household levels. The regressions used linear probability models for both continuous and binary variables for ease of interpretation, unless otherwise specified. Impacts were considered statistically significant at *P* < 0.05. Robustness analysis included estimating treatment effects using fixed-effect regressions with SEs clustered at village level, as well as examining treatment effects on absolute height deficit alongside HAZ (30). Because the allocation of clusters to study arms was random, following Hayes and Moulton (31) we described the magnitude of differences in baseline characteristics across intervention groups, and significance tests of these differences at baseline were not undertaken.

## Results

### Trial attrition

A total of 2626 households in 116 communities were surveyed at baseline in June, 2013. Twenty-five schools in the study population, including ∼18% of children in the target age group (5–15 y), received some form of free school meals at baseline and were removed from the study population (based on the response to a question on whether the school was involved in the GSFP at baseline). Two communities could not be surveyed at end line in March, 2016 due to insecurity problems. The end line survey included 1668 households and 3170 children in 91 communities, leading to an attrition rate of 8%. No statistically significant differences in means of HAZ or BAZ between attrited and nonattrited children were found at baseline (**Supplemental Table 1**). The attrition rate was not significantly different across treatment groups nor was the probability of attrition correlated with treatment assignment (not reported).

### Baseline characteristics and tests of balance

At baseline, the mean household size was 7 members and ∼1 in 5 households were female-headed. Children were, on average, 8.5 y old, and ∼48% of them were girls. School enrollment levels were high at 98%. Overall, no substantive differences between the intervention and control groups were found in the baseline characteristics of the study population (**Table 1**).

**TABLE 1:** Characteristics of the study population at baseline in treatment and control communities, in Ghana, HGSF study^1^

Variables	Control (*n *= 1483)	Intervention (*n *= 1650)	Difference in values^2^
Age, y	8.40	8.54	−0.14
Child is female	0.46	0.49	−0.03
HAZ	−1.11	−1.05	−0.06
BAZ	−0.68	−0.65	−0.02
Child is enrolled in school	0.99	0.98	0.01
Child is enrolled in private school	0.10	0.11	−0.01
Child lives in northern regions	0.43	0.49	−0.06
Household head education, y	3.59	3.84	−0.26
Household head age, y	44.07	45.43	−1.36
Household expenditures, log	7.53	7.52	0.00
Household size, *n*	6.77	6.62	0.16
Dependency ratio^3^	2.03	1.98	0.05
Polygamous household	0.01	0.01	0.00
Female-headed household	0.19	0.20	0.00
Urban	0.06	0.06	0.00

1 All unadjusted baseline values are means or proportions (*n*/*N*). BAZ, BMI-for-age *z* score; HAZ, height-for-age *z* score; HGSF, Home-Grown School Feeding.

^2^Unadjusted absolute value of difference between means.

^3^The dependency ratio was calculated at household level by dividing the number of children aged 0–18 y by the number of adults.

### Uptake of the intervention

Despite high levels of enrollment and low levels of absenteeism in the study population, overall uptake of the intervention was 61% in the intervention group. When restricting the sample to children in the intervention communities who were enrolled in public primary schools (and not private schools that are ineligible for the program), uptake increased to 83%, indicating that most children in public basic education received the school meals intervention. For those children in the intervention group who received school meals, the school meal service was delivered, on average, on 4.6 out of the 5 previous school days, suggesting that the program was functioning regularly. Analysis of the correlates of uptake of the school meals intervention indicated that children aged 5–8 y at baseline were ∼3 times more likely to receive school feeding than those aged 9–15 y at baseline (**Supplemental Table 2**), in line with the expectation that older children progressing to secondary school or being out of school at end line would be less likely to receive the school meals intervention. Girls were no more likely than boys to receive the intervention, whereas children living in the northern regions were ∼5 times more likely to receive school meals than were their counterparts living in the southern regions. Seventy-nine percent of children that received school meals in the treatment arm at end line reported receiving meals during all of the 5 previous school days. Twenty-three percent of chilThe interventiondren in the treatment group reported they were more likely to eat less food at home on days they eat at school, suggestive of potential substitution between meals at home. Only 4% reported bringing food home from their school meal to share with their siblings.

The analysis of the survey data from the school caterers (*n* = 55) found that 86% had experienced irregular payments and approximately one-third of them had not received payment in the 3 mo before the survey (not reported). Approximately 85% of caterers also indicated that payments were often insufficient to cover operational costs, resulting in having to resort to credit to avoid changing the quality of meals (83%), reducing portion sizes (9%), or adopting other strategies to reduce costs.

### Impact on anthropometry indexes

In the 5–15 y population in both treatment and control groups, both HAZ and BAZ declined during the study period. School meals had no effect on HAZ and BAZ in children aged 5–15 y (**Table 2**). However, important heterogeneities in the effectiveness of the intervention by age, gender, household poverty, and geographic location were found in the subgroup analysis following protocol Table 2 and **Tables 3** and **4**. In children aged 5–8 y, school meal provision increased HAZ by 0.12 SDs, whereas no effect of the intervention was found in children aged 9–15 y. Disaggregating the results by gender showed that school meals increased HAZ in school-age girls by 0.11 SDs, and BAZ only in boys aged 5–8 y by 0.19 SDs. In boys aged 9–15 y, school feeding reduced HAZ by 0.18 SDs (*P* = 0.047), although no other negative effects were found in any relevant subgroups for this age cohort.

**TABLE 2:** Unadjusted mean HAZ and BAZ at baseline and after 3 y in the intervention and control groups, and adjusted ANCOVA estimates for these indicators, in children aged 5–15 y at baseline, and by subgroups aged 5–8 y and 9–15 y at baseline living in treatment and control communities in Ghana, HGSF study^1^

		Control				School feeding						
		Baseline		End line		Baseline		End line		ANCOVA		
Age range		Mean	*n*	Mean	*n*	Mean	*n*	Mean	*n*	Impact	SE	*P*
5–15 y	HAZ	−1.11	1354	−1.21	1020	−1.05	1540	−1.12	1165	0.05	0.04	0.298
	BAZ	−0.68	1374	−0.87	1012	−0.66	1551	−0.80	1148	0.08	0.06	0.158
5–8 y	HAZ	−0.96	760	−1.13	601	−0.89	841	−0.97	667	0.12	0.06	0.043
	BAZ	−0.59	769	−0.85	592	−0.53	845	−0.71	649	0.11	0.07	0.115
9–15 y	HAZ	−1.30	575	−1.33	410	−1.25	682	−1.33	489	−0.05	0.06	0.469
	BAZ	−0.79	580	−0.89	409	−0.81	688	−0.91	490	−0.01	0.07	0.931

1 All unadjusted baseline and end line values are means. BAZ, BMI-for-age *z* score; HAZ, height-for-age *z* score; HGSF, Home-Grown School Feeding.

Disaggregating the results by poverty status highlighted a positive effect of school meals on HAZ in children from poor households aged 5–8 y of 0.21 SDs, nearly twice the effect size observed in the 5–8 y population (Table 4). No heterogeneities by gender were found for effects in poor households (not reported).

**TABLE 3:** Unadjusted mean HAZ and BAZ at baseline and after 3 y in the intervention and control groups, and adjusted ANCOVA estimates for these indicators, in children aged 5–15 y at baseline by gender, and by subgroups aged 5–8 y and 9–15 y at baseline living in treatment and control communities in Ghana, HGSF study^1^

		Control				School feeding						
		Baseline		End line		Baseline		End line		ANCOVA		
Subgroup		Mean	*n*	Mean	*n*	Mean	*n*	Mean	*n*	Impact	SE	*P*
Girls	HAZ	−1.09	616	−1.15	454	−0.99	768	−0.97	545	0.12	0.05	0.021
	BAZ	−0.71	628	−0.84	455	−0.64	771	−0.80	536	0.04	0.07	0.535
5–8 y	HAZ	−1.02	352	−1.08	275	−0.92	431	−0.88	332	0.11	0.07	0.103
	BAZ	−0.66	360	−0.85	274	−0.55	433	−0.81	321	0.05	0.10	0.619
9–15 y	HAZ	−1.17	253	−1.25	175	−1.09	325	−1.09	210	0.13	0.09	0.122
	BAZ	−0.78	255	−0.82	176	−0.75	326	−0.76	212	0.03	0.09	0.741
Boys	HAZ	−1.13	738	−1.26	566	−1.10	801	−1.26	642	−0.03	0.07	0.672
	BAZ	−0.65	745	−0.89	556	−0.67	812	−0.79	635	0.08	0.07	0.206
5–8 y	HAZ	−0.90	408	−1.17	326	−0.85	424	−1.05	345	0.10	0.09	0.228
	BAZ	−0.53	409	−0.85	318	−0.51	427	−0.60	338	0.17	0.08	0.028
9–15 y	HAZ	−1.40	322	−1.40	235	−1.40	372	−1.52	289	−0.18	0.09	0.047
	BAZ	−0.79	325	−0.94	233	−0.84	377	−1.02	289	−0.03	0.08	0.687

1 All unadjusted baseline and end line values are means. BAZ, BMI-for-age *z* score; HAZ, height-for-age *z* score; HGSF, Home-Grown School Feeding.

**TABLE 4:** Unadjusted mean HAZ and BAZ at baseline and after 3 y in the intervention and control groups, and adjusted ANCOVA estimates for these indicators, in children aged 5–15 y at baseline living in poor households, and by subgroups aged 5–8 y and 9–15 y at baseline living in treatment and control communities in Ghana, HGSF study^1^

		Control				School feeding						
		Baseline		End line		Baseline		End line		ANCOVA		
Poor^2^ households		Mean	*n*	Mean	*n*	Mean	*n*	Mean	*n*	Impact	SE	*P*
All	HAZ	−1.17	311	−1.22	231	−1.15	353	−1.10	271	0.11	0.08	0.210
	BAZ	−0.84	311	−0.96	225	−0.69	355	−0.84	264	0.06	0.09	0.518
5–8 y	HAZ	−1.03	189	−1.20	149	−0.88	182	−0.84	147	0.22	0.09	0.020
	BAZ	−0.78	190	−1.00	141	−0.50	182	−0.72	140	0.09	0.10	0.359
9–15 y	HAZ	−1.34	119	−1.25	81	−1.44	168	−1.40	123	−0.04	0.16	0.791
	BAZ	−0.94	117	−0.90	83	−0.89	170	−0.97	123	−0.04	0.14	0.789

1 All unadjusted baseline and end line values are means. BAZ, BMI-for-age *z* score; HAZ, height-for-age *z* score; HGSF, Home-Grown School Feeding.

2 Poverty status was determined using household expenditures based on the national poverty line of 1314 Ghanaian cedis.

Disaggregating results geographically showed that school meals had no effect on the nutritional status of the aggregate school-age population in the northern regions of Ghana (not reported). However, the intervention increased HAZ by 0.20 SDs in girls living in the northern regions, with the effects appearing to be driven by increases of 0.27 SDs in girls aged 5–8 y (**Supplemental Table 3**).

Robustness analysis using fixed-effect regression models with SEs clustered at village level confirmed the positive effects on HAZ in girls, in children aged 5–8 y from poor households, and in girls living in the northern regions, as well as the positive effects on BAZ in boys aged 5–8 y. The negative effects on HAZ in boys aged 9–15 y were not confirmed in the robustness analysis. Additional robustness analysis using difference-in-difference regressions resulted in treatment effect estimates that were less precise (i.e., had larger CIs) than those estimated with ANCOVA.

## Discussion

This study is, to our knowledge, the first CRCT to evaluate the impact of a large-scale school meal program operating in a lower-middle-income country. The analysis found evidence of effects of the intervention on the physical growth in school-age children. These effects were heterogeneous, depending on age, gender, poverty status, and geographic location. In terms of linear growth, school meals improved HAZ in the early primary school years (effect size: ∼0.1 SD) in girls, in children from households living below the poverty line, and in those living in the northern regions of Ghana (the country's most impoverished areas). The results suggested that the intervention was particularly effective in improving HAZ in children from poor households (effect size: ∼0.2 SDs) and in girls living in the northern regions (effect size: ∼0.3 SDs). The school meals intervention also increased BAZ, but only in boys of early primary school age. The study also found a negative effect of school meals on boys aged 9–15 y, although the result was at the margin of statistical significance (*P* = 0.05) and was not confirmed in the robustness analysis, suggesting this finding was of a spurious nature. Interpreting these results in the context of Ghana, where the prevalence of overweight and obesity in the study population at baseline was ∼2% and <1%, respectively, highlights the potential from a social protection perspective of the school-based intervention to support child nutrition. Because this is the first CRCT involving an intervention implemented in a national program operating at scale, this study provides important insights for policymakers when compared with the existing evidence base on school feeding. Although the findings on HAZ are novel, those on BAZ are consistent with the literature, where a systematic review and meta-analysis found a small significant effect of school feeding on weight (5). That review also found a small nonsignificant effect on height gain (0.38 cm; 95% CI: −0.32, 1.08 cm) from 3 randomized controlled trials.

The effects found on HAZ on children in the early primary school age group highlight potential plasticity of growth before adolescence. Whether these gains in HAZ correlate with subsequent returns in labor and productivity or in reproductive outcomes remains an important question for further research. A sister study to this analysis, focusing on the impact of school feeding on education outcomes in Ghana, found that the intervention improved cognition and learning in school-age children, with improvements concentrated in girls, the poorest children, and children from the northern regions (32). The findings of these 2 studies are suggestive of important complementarities between the multiple potential benefits of school feeding across nutrition and learning (33).

This study has several strengths, including the CRCT design. In addition, the study population was drawn from school-age children across all 10 regions of Ghana, increasing the external validity of the findings and allowing age disaggregation of results. Some important limitations also arose involving the program implementation. Despite efforts by the government to ensure prompt payment to caterers providing school feeding, delays in disbursements led to implementation delays of >1 y and other bottlenecks that will have likely affected the effectiveness of the intervention. It is notable that the treatment effects reported in this analysis were found despite the implementation challenges and the suboptimal uptake of the intervention. Suboptimal service delivery may result in families of eligible children not knowing if a child will receive a meal or not on a given day, which may be a worse situation than having no meal program at all, because parents and children will not have made alternative feeding arrangements. Understanding the links between the quality of school meal program implementation and child-level impacts remains an important area of further research.

In conclusion, this study suggests that school feeding programs can provide a platform to scale-up nutrition interventions at a key stage of the life cycle, with important benefits accruing for more disadvantaged children. However, important heterogeneities in effect sizes highlight some of the nuances and trade-offs involved that will require further investigation.

## Supplementary Material

nxz079_Supplemental_File

## Notes

Supported by the Partnership for Child Development (to AG) through grants from the Bill & Melinda Gates Foundation (to LD) and Dubai Cares (to LD). We acknowledge support from the CGIAR Research Program on Agriculture for Nutrition and Health, led by the International Food Policy Research Institute.

Author disclosures: AG, EA, GF, DA, CA, IO-A, EM, KW, MF, LD, and HA, no conflicts of interest.

The funder of the study had no role in study design, data collection, data analysis, data interpretation, or writing of the report. AG had full access to all the data in the study and had final responsibility for the decision to submit for publication.

Supplemental Tables 1–3 are available from the “Supplementary data” link in the online posting of the article and from the same link in the online table of contents at https://academic.oup.com/jn/.

Abbreviations used: BAZ, BMI-for-age *z* score; CRCT, cluster randomized control trial; EA, enumeration area; GSFP, Ghana School Feeding Program; HAZ, height-for-age *z* score.
