# Supplementary material for: A School Meals Program Implemented at Scale in Ghana Increases Height-for-Age during Midchildhood in Girls and in Children from Poor Households: A Cluster Randomized Trial
Source: J Nutr. 2019 May 17;149(8):1434–42. doi: 10.1093/jn/nxz079 (PMC6686055; doi:10.1093/jn/nxz079)
Supplement: nxz079_Supplemental_File [file nxz079_supplemental_file.pdf]

Supplementary data.

Supplementary material

Supplemental Table 1: Unadjusted means and difference in means at baseline between attrited and non-attrited children for key outcomes and household characteristics in Ghana, HGSF study

Variables	Non-attrited *n*	Non-attrited Mean (prop.)	Attrited *n*	Attrited Mean (prop.)	Difference in values
Age, y	2283	8.22	849	9.16	-0.95
Child is female	2283	0.46	850	0.53	-0.07
HAZ	2019	-1.07	850	-1.10	0.037
BAZ	2056	-0.66	843	-0.67	0.01
Is enrolled in school	2126	0.99	777	0.97	0.02***
Lives in northern regions	2283	0.47	850	0.43	0.03*
Household head education years, y	2283	3.73	850	3.71	0.01
Household head age, y	2269	44.43	841	45.75	-1.31***
Log of household expenditures	2283	7.53	849	7.51	0.02
Household size, n	2283	6.62	850	6.88	-0.26**
Dependency ratio	2278	2.01	848	1.98	0.03
Polygamous household	2283	0.01	850	0.01	0.01
Female-headed household	2283	0.19	850	0.21	-0.01
Urban	2283	0.07	850	0.06	0.018

Notes:[1] All unadjusted baseline values are means or proportions. HAZ, height-for-age z-score; BAZ, BMI-for-age z-score. The dependency ratio calculated at household level by dividing the number of children aged 0-18y by the number of adults. **P<0.10*, ***P<0.05*, ****P<0.001.*

Supplementary data.

Supplemental Table 2: Correlates of program uptake at endline after 3y in the intervention group, in children aged 5-15y at baseline living in treatment communities in Ghana, HGSF study[1]

	Odds Ratio	S.E.	P
Is aged 5-8y	2.91	0.57	<0.001
Child is female	0.76	0.14	0.141
Lives in northern regions	4.20	2.75	0.028
From poor household	1.15	0.33	0.617
In private school	0.05	0.02	<0.001
Household size, n	1.02	0.05	0.650
Female-headed household	0.82	0.25	0.516
Age of household head	1.00	0.01	0.937
Constant	0.74	0.50	0.656
n	1,466		

Notes: [1]Mixed effects logistic regression results with random intercepts at cluster level. S.E., standard error.

Supplementary data.

Supplemental Table 3: Unadjusted mean HAZ and BAZ, at baseline and after 3y in the intervention and control groups, and adjusted ANCOVA estimates for these indicators, in children aged 5-15y at baseline living in the 3 northern regions, and by subgroups aged 5-8y and 9-15y at baseline living in treatment and control communities in Ghana, HGSF study[1]

Sub-group		Control				School feeding				(ANCOVA)		
		BL		EL		BL		EL				
		mean	*n*	mean	*n*	mean	*n*	mean	*n*	Impact	SE	P
Girls (north)	HAZ	-1.14	258	-1.33	188	-1.07	348	-0.95	257	0.22	0.08	0.007
	BAZ	-0.68	263	-0.79	193	-0.70	350	-0.80	252	0.05	0.12	0.652
5-8y (north)	HAZ	-1.06	148	-1.29	111	-0.99	202	-0.86	164	0.27	0.10	0.010
	BAZ	-0.65	151	-0.83	112	-0.60	204	-0.76	159	0.11	0.15	0.433
9-15y (north)	HAZ	-1.24	106	-1.39	76	-1.20	138	-1.10	93	0.15	0.14	0.298
	BAZ	-0.73	107	-0.74	80	-0.89	138	-0.87	93	-0.04	0.18	0.823

[1] All unadjusted baseline and endline values are means. HAZ, height-for-age z-score; BAZ, BMI-for-age z-score. SE., standard error.
